# Management of Teeth with Grade 3 Endo-Periodontal Lesions by Combined Endodontic and Regenerative Periodontal Therapy

**DOI:** 10.3390/jcm13010093

**Published:** 2023-12-23

**Authors:** Christina Tietmann, Ivet Tezer, Emad Youssef, Søren Jepsen, Karin Jepsen

**Affiliations:** 1 Private Practice for Periodontology, 52070 Aachen, Germany; 2 Department of Periodontology, Operative and Preventive Dentistry, University of Bonn, 53111 Bonn, Germany

**Keywords:** endo-periodontal lesion, regenerative periodontal therapy, furcation involvement, periodontal attachment loss, bovine bone mineral, enamel matrix derivative

## Abstract

(1) Background: Severely compromised teeth affected by endo-periodontal lesions are often assigned a “hopeless” prognosis, however, there is only limited evidence available. (2) Methods: In a retrospective study, we evaluated the long-term effectiveness of combined endodontic and regenerative periodontal therapy in teeth with advanced endo-periodontal lesions: 35 patients (age 47–83 years) with a total of 39 teeth diagnosed with grade 3 endo-periodontal lesions were treated by endodontists using an operating microscope followed by regenerative periodontal surgery. (3) Results: Changes in radiographic bone levels (rBl) and probing pocket depths (PPDs) were evaluated after 1 year (T_1_) and up to 7 years postoperatively (T_final_). Mean rBL gain was significant with 4.87 ± 3.47 mm after 1 year (T_1_) and stable results with a mean rBL gain of 4.70 ± 3.37 mm at T_final_. Mean PPD was significantly reduced from 9.74 ± 2.05 mm at baseline to 5.04 ± 1.61 mm at T_1_ and to 4.87 ± 2.32 mm at T_final_. Tooth loss amounted to 10.3% (*n* = 4) and was due to root fracture. (4) Conclusion: The results suggest that the combined endodontic and regenerative periodontal therapy of endo-periodontal lesions of “hopeless” teeth can lead to favorable long-term results with tooth retention for up to 7 years.

## 1. Introduction

Endo-periodontal lesions are defined as “pathologic communication between pulp and periodontal tissues” of a particular tooth appearing in acute or chronic form. They are specified regarding signs and symptoms with a direct impact on prognosis and therapy [1].

Typical signs of an endo-periodontal lesion are deep probing pocket depths extending to the apex of the tooth and altered signs of the pulp vitality. In addition, further signs and symptoms such as radiographic bone loss extending to the apical region of the tooth and furcation involvement, sensitivity to palpation/percussion, increased tooth mobility, bleeding on probing and/or pus suppuration can be apparent [1].

According to the “2017 World Workshop on the Classification of Periodontal and Peri-Implant Diseases and Conditions” they are classified into endo-periodontal lesions with root damage—such as root fracture, perforation of a root canal, root resorption—or without root damage. Endo-periodontal lesions without root damage are further subdivided with regard to periodontitis and non-periodontitis patients as well as three grades of severity [1,2]. Based on this new classification, endo-periodontal lesions have a prevalence of 4.9% [3].

Teeth diagnosed with advanced endo-periodontal lesions are often considered “hopeless” and extracted without any treatment effort and replaced by an implant supported restoration. In light of the increasing prevalence of peri-implant disease, tooth extraction should not be considered as the first treatment option. Instead, tooth retention even for periodontally compromised teeth should be considered as the first treatment choice [4,5,6,7].

There is a common consensus in the recent literature that root canal treatment in teeth affected by endo-periodontal lesions should be performed first [8,9,10]. However, sole endodontic treatment has failed to prove as a highly predictable therapy for teeth affected by endo-periodontal lesions [10]. Therefore, additional periodontal therapy is indicated, in particular, regenerative procedures for grade 3 endo-periodontal lesions presenting with deep periodontal pockets at more than one tooth surface to enhance the outcome and prognosis. The time-lapse between endodontic and regenerative periodontal treatment did not show to have any effect on the healing of endo-periodontal lesions [11,12]. While the value of regenerative therapy in intra-bony defects is well known [6,13,14,15,16,17], only limited data exist on the outcomes of periodontal regenerative treatment of teeth severely compromised by endo-periodontal lesions. So far, there are only a few case reports and series [18,19,20,21] showing favorable results for this combined endodontic and periodontal therapy. Two randomized controlled clinical trials have reported that concomitant regenerative periodontal surgery enhanced the outcomes and success rate after 1 year [12,22]. A 5-year survival rate of 92.31% has been reported in a retrospective study for teeth with endo-periodontal lesions after periodontal regenerative procedures [23].

The aim of this retrospective study was to gain more evidence by evaluating the long-term effectiveness of endodontic treatment followed by regenerative periodontal surgery in teeth with advanced endo-periodontal lesions.

## 2. Materials and Methods

### 2.1. Study Design and Patients

This retrospective study was performed in a periodontal specialty practice (CT) in Aachen and the Department of Periodontology, Operative and Preventive Dentistry (KJ), University of Bonn, Germany. The study was conducted in accordance with the Helsinki Declaration (version 2008) and approved by the Ethics committee of the University of Bonn (#51/22).

Patients were selected during the period from April 2013 to September 2022 if presenting with a grade 3 endo-periodontal lesion according to the 2017 World Workshop on the Classification of Periodontal and Peri-Implant Diseases and Conditions [1]. They were consecutively treated by endodontic specialists under an operating microscope followed by regenerative periodontal surgery using deproteinized bovine bone mineral with/without collagen membrane and/or enamel matrix derivative. Data of these patients were available for a retrospective analysis of the outcomes of this combined treatment. Patients had given their written informed consent for a retrospective evaluation of their clinical and radiographic data.

Inclusion criteria for the analysis were as follows:

- Grade 3 endo-periodontal lesion;
- Endodontic treatment was performed first and followed by periodontal regenerative surgery;
- One- or two-wall intra-bony defects suitable for periodontal regenerative surgery;
- Adequate oral hygiene and control of inflammation: full-mouth plaque score (FMPS) of ≤20% and full-mouth bleeding score (FMBS) of ≤20%;
- Clinical and radiographic data of a consecutive follow-up of at least 1 year after regenerative therapy.
- Exclusion criteria for the analysis were as follows:
- Non-compliance;
- Incomplete probing or radiographic data;
- Heavy smokers > 10 cigarettes per day;
- Resective periodontal surgery on target tooth;
- Teeth with fractures, external or internal resorption;
- Additional periodontal or maxillofacial treatment in the areas of interest;
- Systemic diseases (e.g., uncontrolled diabetes).

Patients with systemic diseases (i.e., controlled diabetes) were not excluded. Based on the criteria above, 35 patients with a total of 39 endo-periodontal lesions were included in the analysis. All patients were periodontitis patients except for 2 patients. Patient and defect characteristics are depicted in Table 1.

### 2.2. Treatment

#### 2.2.1. Endodontic Therapy

All endodontic treatments were performed under local anesthesia and sterile conditions by application of rubber dam by three different endodontic specialists (EY, Bonn; PD, Aachen; VK, Aachen). Instrumentation was performed by hand and rotary files (Reciproc^®^, VDW GmbH, Munich, Germany) using a surgical operating microscope (ProErgo, Carl-Zeiss AG, Jena, Germany). All root canals were irrigated intermittently with sodium chloride solution (NaCl) as well as 3% sodium hypochloride solution (NaOCl). At the end of the first appointment, calcium hydroxide paste was applied into all canals. In a second appointment, at least 2 weeks later, obturation of all root canals was achieved either by thermoplastic filling or lateral condensation techniques using gutta-percha. The cavity was sealed with a resin-bonded composite material. Periapical radiographs were taken prior, during and after completion of the endodontic treatment.

#### 2.2.2. Regenerative Periodontal Surgery

Regenerative periodontal therapy of the selected areas was performed after a mean re-evaluation time of three months after endodontic treatment by two periodontists (CT, Aachen and KJ, Bonn) if the lesion still persisted.

Following administration of local block and infiltration anesthesia, a minimally invasive microsurgical approach for incisions and flap was chosen as previously described [14]. For optimal wound stability and to maintain better blood supply of the flap, vertical releasing incisions were avoided by extending flap design to adjacent teeth.

Apical split flap preparation was only performed if needed for primary tension-free flap closure. After meticulous debridement of the defect with curettes, sonic/ultrasonic devices the biomaterial was selected depending on the configuration of the intra-bony defect [6,13,26]. To avoid a soft-tissue collapse into the defect, a bone filler was used (DBBMc, Bio Oss^®^ Collagen; Geistlich, Wolhusen, Switzerland). If the graft material was at risk for dislocation in non-contained defects, a collagen membrane (Bio Gide^®^Perio; Geistlich, Wolhusen, Switzerland) was applied without pin or suture fixation. Enamel matrix derivative (EMD, Emdogain^®^; Straumann, Basel, Switzerland) was applied in contained defects after debridement as an adjunct to the root surface. To achieve primary tension-free closure of the coronally positioned flap, modified horizontal mattress sutures and additional single interrupted sutures for papilla adaptation (Premilene^®^ USP6/0-DS13, B. Braun, Tuttlingen, Germany; Seralene^®^ USP6/0-DS12 SeragWiessner, Naila, Germany; Seralene^®^USP6/0-DS15; SeragWiessner, Naila, Germany) were used.

Postoperatively, a strict anti-infective protocol was enforced instructing the patient to rinse with 0.12% chlorhexidine solution three times a day and to refrain from mechanical tooth cleaning in the surgically treated areas for 2 weeks or until complete wound healing.

Sutures were removed after 10–14 days depending on individual wound healing progression. In the case of advanced tooth mobility > grade I (Miller, 1938) [27], a removable acrylic splint was inserted after surgery to provide wound stability of the regeneratively treated site.

#### 2.2.3. Supportive Periodontal Therapy

Following periodontal surgery, supportive periodontal therapy started with a strict interval of 4 weeks up to 3 months. Control of inflammation was accomplished by cautious professional tooth cleaning and oral hygiene reinforcement according to the individual patient needs with an average of three times per year.

### 2.3. Clinical and Radiographic Assessments

**Figure 1:**
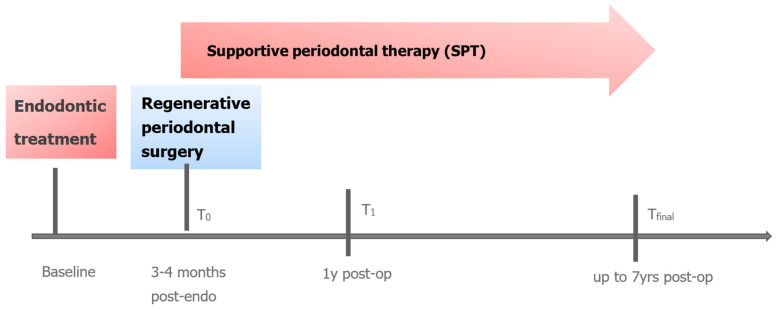
Chronical sequence of treatment and examinations (T_0_ = baseline, T_1_ = 1 year, T_final_,).

Periodontal measurements for all 39 lesions were taken at baseline (T_0_) before periodontal regenerative surgery with a mean observation time of 3.9 months after endodontic therapy, after 1 year (T_1_) and every year during supportive care up to the 7-year follow-up visit (T_final_) using a periodontal probe (PCP11; Hu-Friedy) (Figure 1):

- Probing pocket depth (PPD) at 6 sites per tooth;
- Full-mouth plaque scores (FMPS), Full mouth bleeding scores (FMBS);
- Horizontal furcation class (0-III) (Hamp et al., 1975) [24] and vertical furcation subclasses A, B, C (Tarnow and Fletcher, 1984) [25];
- Tooth mobility (Miller, 1938) [27].

The tooth site with the most advanced bone loss mesially or distally (distance between the cemento-enamel junction or restoration to the bottom of the defect) as measured during surgery became the target site. Intra-operative BL was used for calibration of the pre-operative periapical radiograph by using the overall tooth length as a reference length, as previously described [14]. All radiographs were analyzed using ImageJ Software (Version 1.43u, National Institutes of Health, Bethesda, MD, USA) by a trained and calibrated examiner (IT) who was not involved in the surgeries. 

### 2.4. Statistical Analysis

Change of radiographic bone level (rBL) was the primary outcome parameter, whereas change in PPD and frequency of pocket closure (sites with PPD ≤ 4 mm) served as secondary outcomes. Descriptive analysis was outlined for radiographic bone level (rBL) and pocket probing depth (PPD) with change over time at three time points T_0_, T_1_, T_final_ using means and standard deviations per tooth.

The comparisons of radiographic bone level between time points were performed with a Wilcoxon signed-rank test. To take into account a possible clustering on patient level, a Wilcoxon signed-rank test for clustered data (Rosner et al., 2006) [28] was used with the method developed by Datta and Satten (2008) [29].

To compare the proportion of pocket closures between time points, a logistic regression with time as the fixed effect and tooth-id and, if necessary, patient as the random effect was run. The *p*-values for the comparison with pocket closure at baseline were adjusted with an approximation of the Dunnet-correction, while for the pairwise comparison between all three time points, a Tukey correction was used.

Although the data was of two level-nature (patient–tooth), only a few patients (n = 3) had in fact more than one tooth in this study. Hence, to investigate the impact of BOP, FMPS and smoking on RBL and PPD, a two-level model with patient as random factor was used if indicated. In all other cases, the random factor was dropped and a linear regression was used instead.

The effect of occlusal wear on tooth loss was investigated by running a logistic regression on tooth loss with occlusal wear as a factor. Adding patient as a random factor resulted in a near-singular fit due to the near zero variation within patient; however, both models showed the same estimates. Level of significance was set for *p* < 0.05.

Statistical analyses of the clinical and radiographic data were performed by an independent expert biostatistician with the statistical software R, version 4.1.3 (R Core Team, 2022) [30].

## 3. Results

### 3.1. Patient and Defect Characteristics

A total of 35 patients (Aachen, 28 patients; Bonn, 7 patients) with 39 teeth diagnosed with an endo-periodontal lesion were included in the study. All patients showed a history of periodontitis except for two patients. Three patients contributed more than one treated lesion. In total, 24 teeth were molars including 18 molars (11 mandibular and 7 maxillary molars) presenting with horizontal furcation class I (n = 4), class II (n = 13) and class III (n = 1) (Hamp et al., 1975) [24]. According to the vertical subclassification of molars with class I-III furcation (Tarnow and Fletcher, 1984) [25], there were 2 molars presenting with subclass A, 3 with subclass B and 13 with subclass C. Six molars did not show any furcation involvement (Table 1).

For all 39 teeth treated by the combined therapy of endodontic treatment and regenerative periodontal surgery, data were available 1 year postoperatively (T_1_). Data of a longer follow-up of up to 7 years (T_final_) with a mean observation time of 31.4 months-could be obtained for 23 teeth in 23 patients.

All of the patients showed full adherence to supportive care during the observation period. At baseline, the mean radiographic bone level was 12.54 ± 3.63 mm with a mean PPD of 9.21 ± 2.15 mm. For the 23 teeth with complete follow-up data, the baseline values were a mean rBL of 13.09 ± 3.12 mm with a mean PPD of 9.74 ± 2.05 mm (Table 1 and Table 2a–c).

### 3.2. Outcomes

Surgeries and soft tissue healing were generally uneventful with none of the patients developing any major complications or allergic reactions, suppuration or abscesses. Minor complications such as postoperative swelling and pain in the surgically treated area resolved within a few days after surgery.

**Figure 2:**
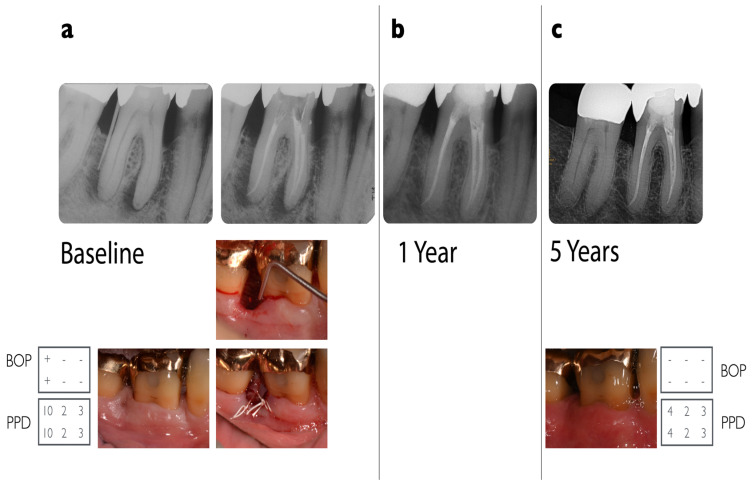
Radiographic and clinical situation of a lower right molar (#46) in a 55-year-old otherwise healthy patient. Five-year follow-up of a grade 3 endo-periodontal lesion treated with combined endodontic and regenerative periodontal therapy. (**a**) The patient came for his regular 3-monthly re-evaluation visit for his periodontitis stage III. Deep probing depths at tooth #46 distal of 10 mm (visualized by a gutta-percha point) not evident at his last visit. The furcation was not involved. Radiograph with evidence of extensive vertical and peri-apical bone loss. Pulp sensitivity testing was negative when exposed to chlorethylene and to electric (Vitality Scanner™) scanning. Root canal treatment was performed under an operating microscope. For the regenerative procedure, a papilla preservation flap technique was performed, using DBBM (BioOss-Collagen^®^Geistlich) and EMD (Emdogain^®^, Straumann, Basel, Switzerland) as supportive materials. Healing was uneventful, and monofilic sutures (6/0) were used. (**b**) One-year follow-up with no evidence of radiographic pathologies. (**c**) Five-year follow-up after regenerative surgery: probing depths: 2–4 mm; recession: up to 3 mm, with a radiograph showing stable radiographic bone fill in the vertical defect area.

**Figure 3:**
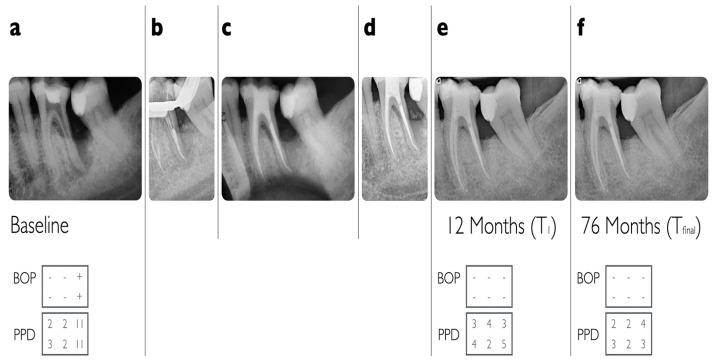
(**a**) Radiographic situation of a lower left molar (#36) in a 47-year-old otherwise healthy patient; (**b**) endodontic treatment/downpack; (**c**) radiographic control immediately after endodontic treatment; (**d**) 3 months after endodontic treatment: radiographic improvement but still persisting deep probing pocket depths (food impaction visible); (**e**) at 12-month follow-up (T_1_); (**f**) at 76-month follow-up (T_final_).

Two representative examples of teeth affected by advanced endo-periodontal lesions that were included in the present analysis are illustrated in Figure 2 and Figure 3.

**Figure 4:**
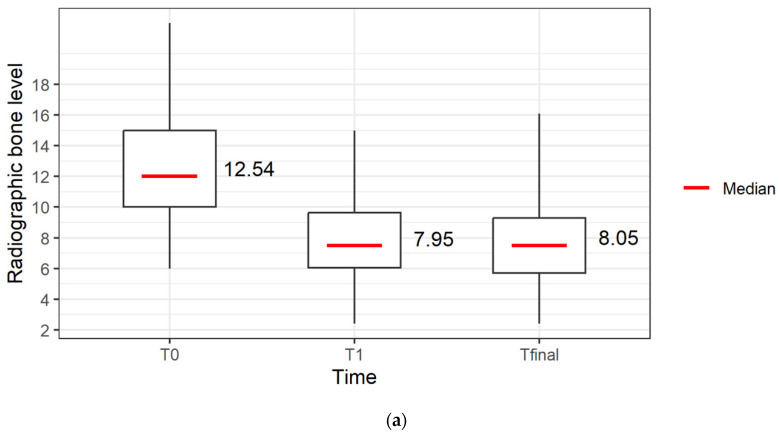

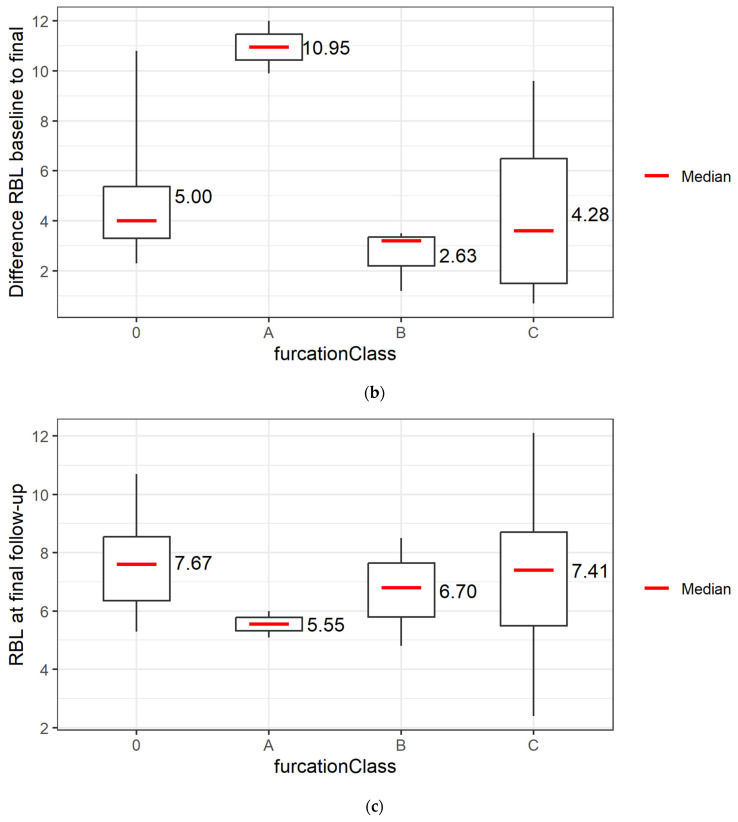
(**a**) Radiographic bone level over time (T_0_ = baseline), 1 year after regenerative surgery (T_1_) and up to 7 years after regenerative therapy (T_final_) of 39 teeth in 34 patients. (**b**) Radiographic bone level gain and (**c**) radiographic bone level at T_final_ of 24 molars in 23 patients without furcation involvement (*n* = 6) or vertical furcation subclasses A (*n* = 2), B (*n* = 3), C (*n* = 13) from T_0_ = baseline and of 21 molars in 20 patients up to 7 years after regenerative surgery (T_final_) without furcation involvement (*n* = 6) or vertical furcation subclasses A (*n* = 6), B (*n* = 11), C (*n* = 0), and 3 molars lost due to root fracture.

Radiographic bone level improved significantly from 12.54 ± 3.36 mm at baseline to 7.95 ± 2.65 mm after 1 year *(p* < 0.0001). Analysis of change in radiographic BL over time of 23 teeth with available data over all time points revealed a statistically significant mean gain in radiographic bone level of 4.8 ± 3.47 mm (*p* < 0.0001) at T_1_ and 4.70 ± 3.37 mm at T_final_ (*p* < 0.0001) (Figure 4a and Table 2a,b).

Mean PPD was significantly reduced from 9.21 ± 2.15 mm at baseline to 5.21 ± 1.79 mm after 1 year. For 23 teeth with available data over all time points, a significant reduction of 4.70 ± 2.42 mm (*p* < 0.0001) at T_1_ was achieved and remained stable with 4.87 ± 2.93 mm at T_final_ (*p* < 0.0001) (Table 2c).

Pocket closure could be achieved in 34.8% after 1 year and 52.2% at T_final_ and was statistically significant (Table 2d,e).

Fourteen out of eighteen molars with horizontal furcation involvement showed an improvement by conversion to a lower furcation class, revealed by a decrease in the frequency of horizontal furcation class II of 54% at baseline to 13% and an increase in the frequency of furcation classes I and 0 to 46% and 42%, respectively, after 1 year. For molars (*n* = 16) with available data for all three time points, the horizontal furcation class remained stable from T_1_ to T (Table 3a).

If the 18 molars affected by horizontal furcation involvement were subdivided according to vertical furcation subclasses, the change in rBL at T_final_ showed a gain of 10.95 mm in vertical subclass A (*n* = 2, 11%), a gain of 2.63 mm in subclass B (*n* = 3, 17%) and a gain of 4.28 mm in subclass C (*n* = 13, 72%) with a final radiographic bone level of 5.55 mm in subclass A, 6.70 mm in subclass B and 7.41 mm in subclass C (Table 3b and Figure 4b,c).

Overall, 89% of the furcations showed improvement in vertical subclassification throughout the study period. All vertical subclasses C were resolved at T_1_, showing a conversion to subclass B (56%) or to subclass A (17%) and stable results at T_final_ (Table 3b).

**Figure 5:**
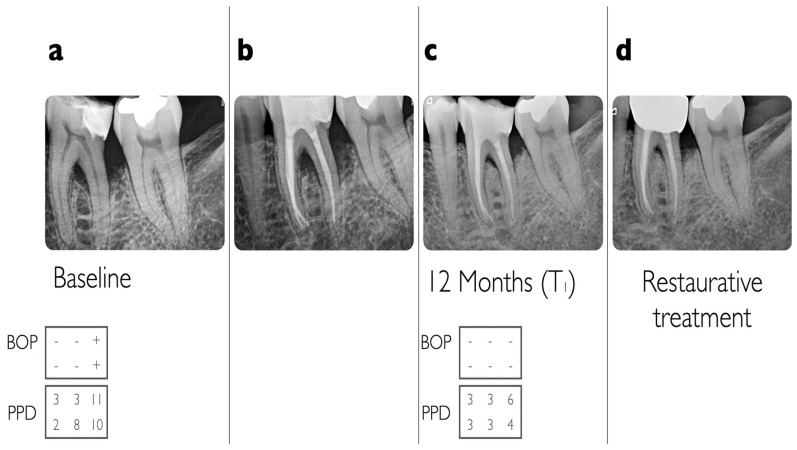
(**a**) Baseline radiographic situation of a lower right molar (#36) with horizontal furcation involvement class III with vertical furcation subclass B component, (**b**) 3 months after endodontic treatment, (**c**) at 12-month follow-up (T1) with conversion to horizontal furcation class I, and (**d**) after restorative treatment.

A representative example of teeth affected by advanced endo-periodontal lesions with vertical subclass that were included in the present analysis is illustrated in Figure 5.

No tooth loss was observed after one year, whereas at T_final_ tooth loss amounted to 10.3%, namely four teeth (one premolar and three molars) in three patients due to root fracture; three of them were in patients with bruxism. All three molars that were lost—two maxillary and one mandibular molar—had presented with vertical subclass C at the time of surgery, two of them with horizontal furcation class II and one with class I.

Due to the low proportion of teeth per patient, a multilevel analysis could only be run as a simple one-level regression model, demonstrating a slight impact of rBL at baseline on the outcomes, whereas smoking and BOP did not show any effect on the outcomes.

The results of the final model are presented as Table S1 in the Supplementary Materials.

## 4. Discussion

The data of this retrospective clinical cohort study reveal the long-term effectiveness of a combined endodontic and periodontal regenerative treatment in patients with advanced endo-periodontal lesions. Teeth diagnosed as “hopeless” due to advanced endo-periodontal lesions could be retained in 89% up to 7 years using the combination of endodontic and regenerative periodontal therapy. These findings are novel and of high clinical relevance.

The results of this investigation cannot be easily compared to previously published studies due to differences in study protocols with regard to regenerative procedures, performed endodontic treatment, baseline clinical severity, selection of outcome measures and length of follow-up.

A recent retrospective long-term study has shown that root canal treatment is a valid option for long-term tooth survival with 97% to 68% after 10 and up to 37 years [32]. However, the presence of endo-periodontal lesions with deep periodontal pockets ≥ 6 mm and the presence of periapical radiolucencies [32], as well as furcation involvement [1,33], seem to have a negative impact on long-term prognosis. Thus, the slightly lower retention rate of 89.7% for up to 7 years in our study on teeth with endo-periodontal lesions is probably due to the complexity of pre-existing risk factors such as furcation involvement and advanced bone destruction of the affected teeth [1,32,33]. Interestingly, the results are similar to the 92.3% tooth retention observed in a retrospective study on the combined treatment of endo-periodontal lesions after 5 years [23]. In that study, 45 out of 52 regeneratively treated teeth had received endodontic prior to regenerative periodontal treatment [23]. With regard to the mean radiographic bone level gain, the authors reported 1-year data. Their findings of a rBL gain of 5.30 mm and PPD reduction of 4.44 mm after 1 year (teeth with endodontic treatment in combination with bovine mineral with/without membrane) are similar to the outcomes of our study (4.87 mm of rBL gain and 4.7 mm PPD reduction).

The achieved bone level gain in the present study compares well to the outcomes of 3.9 mm mean bone level gain 1 year after regenerative surgery that remained stable for 10 years observed in a former study performed with a similar regenerative protocol in the same practice [14]. In contrast to the present study, however, no endo-periodontal lesions were included.

With regard to the complexity of defect morphology and severity of risk factors, three studies may serve for comparison for the retention of “hopeless” teeth, even though they did not focus specifically on endodontic-periodontal lesions [4,5,34]. Cortellini et al. (2011, 2020a) [4,5] in a prospective study reported 5- and 10-year follow-up data of teeth initially presenting with attachment loss to the apex that had been treated using regenerative periodontal surgery. In their study, 20 out of 25 teeth had been endodontically treated, 12 teeth associated with defects extending beyond the apex were endodontically treated as a prophylactic measure prior to surgery and eight teeth already presented with previous endodontic treatment. However, it has to be emphasized that one major difference to our study was that only teeth with clearly detectable peaks of bone/attachment on the adjacent teeth were included in these studies, thus enabling a higher radiographic bone level gain of 8.5 ± 3.1 mm in comparison to our outcomes of 4.87 ± 3.47 mm after 1 year in periodontitis patients. Moreover, the initial mean radiographic bone level (16.00 ± 2.3 mm) was greater than 13.09 ± 3.12 mm in our study and therefore adding to the possibility of a higher radiographic bone level gain. The same applies to the possibility of a higher PPD gain: mean PPD gain after 1 year with 4.31 mm (baseline PPD 9.74 mm with 4.87 mm at T_final_) in our study was less than data in the named study with 8.7 mm. In addition, PPD at baseline (12.7 mm) in the study of Cortellini et al., 2011, 2020a [4,5], was much higher than in our study (9.74 mm), thus more favorable for a higher PPD gain. However, our findings of residual probing pocket depths (5.04 ± 1.61 mm after 1 year vs. 4.0 ± 1.7 mm) and tooth retention (89.7% after up to 7 years vs. 88% after 10 years) compare well with the results by Cortellini et al. (2011, 2020 a) [4,5].

A retrospective analysis evaluated the results of periodontal regeneration of 49 molars severely compromised by combined furcation and intra-bony defects [34]. Here, 10 molars had received prophylactic endodontic treatment prior to regenerative surgery. Besides the improvement in horizontal furcation involvement, furcation vertical subclassification improved 1 year after periodontal regenerative surgery in 87.5% (maxillary) and 84.6% (mandibular) of molars, respectively, which compares well to the observed 89% improvement in vertical furcation subclass in the present study. The same applies to the survival rate based on vertical subclassification: the findings of 100% at 1-year follow-up and 88% up to 7 years compare well to the reported survival rates ranging from 100% after 1 year and 95% after an average follow-up of 5.7 years in the study of Cortellini et al. (2020b) [34].

Previous studies have indicated [15,33] that the severity of furcation involvement and vertical subclassification seem to be an important predictor of tooth survival. A shift of a vertical subclass C or B to a subclass A or B can reduce premature extraction of teeth diagnosed with “hopeless prognosis” and prolonging the time of a more costly implant placement or tooth-supported fixed partial denture [5,33].

Therefore, the improvement in vertical subclasses, as shown in the present study by the elimination of subclass C and shift to subclass B (56%), respectively, and subclass A (17%) at T_1_ by regenerative surgery, indicates an increased periodontal support and thus a more favorable long-term prognosis. In addition, the improvement to horizontal furcation class I and 0 after 1 year (46% and 42%) and stable conditions for up to 7 years also add to a better long-term prognosis [15,35,36]. These findings—the improvement in bone level in different furcation subclasses in regeneratively treated molars with endo-periodontal lesions—are novel and of high clinical relevance.

Pocket closure could be achieved in 34.8% after 1 year and 52.17% at T_final_. The rather low proportion of pocket closure is due to the severity of bone loss in teeth diagnosed with endo-periodontal lesions, and therefore, a PPD reduction of more than 50% can be considered a successful treatment in endo-periodontal lesions [10]. Tooth loss of 10.3%, due to root fracture as a consequence of compromised tooth structure and to occlusal wear (75%), is in agreement with the literature [32].

The gain in radiographic bone level and probing pocket depth reduction indicate that even severely compromised teeth diagnosed with endo-periodontal lesions can be retained for a long period of time by the treatment protocol presented in the present study.

The present study has strengths and limitations. Adding to the strengths, all patients were treated by the same two experienced surgeons, and postoperative data measurements and radiographs were evaluated by the same blinded examiner, who was not involved in the clinical evaluation. Data analysis was performed by an independent expert statistician. Furthermore, the study was accomplished independent from industrial support. However, the present study also has inherent limitations due to its retrospective character and the lack of a comparison group. Therefore, it has to be regarded as an extensive feasibility study that may be of great value for the design of future prospective randomized clinical trials.

In the future, well planned prospective randomized controlled clinical trials will further refine the most suitable protocol for the combined endodontic and regenerative periodontal treatment of severely compromised teeth with endo-periodontal lesions.

## 5. Conclusions

Within the limitations of the retrospective study design, the results of this study indicate that the combined therapy of endo-periodontal lesions of “hopeless” teeth with endodontic and regenerative periodontal therapy followed by a strict maintenance protocol can lead to favorable long-term results for tooth retention up to 7 years.

## Figures and Tables

**Table 1:** Patient demographic and defect characteristics at baseline (T_0_).

Patient Demographic Characteristics (*n* = 35)	
Female gender	22/62.9%
Male gender	13/37.1%
Age (mean ± SD/range)	63.7 ± 9.06 (47–83 yrs)
Smokers	8/22.8%
Time between endodontic and regenerative therapy (mean ± SD month)	3.89 ± 3.84
**Defect characteristics (*n* = 39)**	
Single-rooted	13
Multi-rooted (2 premolars, 24 molars)	26
Mandibular molars with/without furcation involvement	11/6
Maxillary molars with/without furcation involvement	7/0
Molars with horizontal furcation class I/II/III *	4/13/1
Molars with furcation involvement—subclass A**	2
Molars with furcation involvement—subclass B **	3
Molars with furcation involvement—subclass C **	13
Probing pocket depth at deepest site (mean ± SD/mm)	9.21 ± 2.15
Bone level (CEJ to bottom of defect intrasurgically) at deepest site (mean ± SD/mm)	12.54 ± 3.44
Follow-up time (mean ± SD / months) baseline	31.41 ± 21.92
Occlusal wear (teeth %)	48.7%
Full Mouth Bleeding Score (mean ± SD/%)	23.16 ± 16.67
Full Mouth Plaque Score (mean ± SD/%)	28.80 ± 13.30

* horizontal furcation class 0–III (Hamp et al. 1975 [24]), ** vertical furcation subclass A, B, C (Tarnow and Fletcher, 1984 [25]).

**Table 2:** (**a**) Mean radiographic bone level (rBL) ± standard deviation (mm) at different time points. (**b**) *p*-values for testing rBL between time different points (statistics Wilcoxon signed-rank test from the R-library coin version 1.3.1 (Hothorn et al., 2008)) [31]. (**c**) Mean probing pocket depth (PPF) ± standard deviation (mm) at different time points. (**d**) Frequency distribution of residual PPD. (**e**) *p*-values for testing proportion of pocket closure (pc) between time points corrected for multiple testing (Tukey test).

(**a**)					
		***N***	**T_0_**	**T_1_**	**T_final_**
rBL per tooth		39	12.54 ± 3.36	7.95 ± 2.65	NA
rBL per tooth with final follow-up > 12 months		23	13.09 ± 3.12	8.21 ± 3.08	8.39 ± 3.70
(**b**)					
			** T_0_.T_1_**	** T_0_.T_final_**	** T_1_.T_final_**
rBL per tooth for all 39 teeth			1.00 × 10^−7^	1.00 × 10^−7^	NA
rBL per tooth for 23 teeth with final follow-up > 12 months			3.35 × 10^−5^	3.83 × 10^−5^	0.78
(**c**)					
		***N***	**T_0_**	**T_1_**	**T_final_**
PPD per tooth		39	9.21 ± 2.15	5.21 ± 1.79	NA ±
PPD per tooth with final follow-up > 12 months		23	9.74 ± 2.05	5.04 ± 1.61	4.87 ± 2.32
(**d**)					
** PPD (mm) **	** Baseline T_0_**	** T_1_**		** T_final_**	
≤4	0 (0.00%)	8 (34.78%)		12 (52.17%)	
5	0 (0.00%)	6 (26.09%)		4 (17.39%)	
6	0 (0.00%)	4 (17.39%)		2 (8.70%)	
7	2 (8.70%)	4 (17.39%)		2 (8.70%)	
8	4 (17.39%)	1 (4.35%)		1 (4.35%)	
9	4 (17.39%)	0 (0.00%)		0 (0.00%)	
10	5 (21.74%)	0 (0.00%)		2 (8.70%)	
11	1 (4.35%)	0 (0.00%)		0 (0.00%)	
12	7 (30.43%)	0 (0.00%)		0 (0.00%)	
(**e**)					
comparison	*p*-value				
pc0–pc1	0.0122				
pc0–pcfinal	0.0050				
Pc1–pcfinal	0.3742				

T_0_ = baseline, T_1_ = 1 year, T_final_ = up to 7 years.

**Table 3:** (**a**,**b**). Horizontal and vertical furcation subclasses at different timepoints, conversion of horizontal and vertical furcation subclass.

(**a**)			
Molars with/without horizontal furcation class (*n* = 24) *	** T_0_**	** T_1_**	** T_final_**
	*N* = 24	*N* = 24	*N* = 16
0	6 (25%)	10 (42%)	7 (44%)
I	4 (17%)	11 (46%)	7 (44%)
II	13 (54%)	3 (13%)	2 (12%)
III	1 (4%)	0 (0%)	0 (0%)
**Conversion of horizontal furcation class**		*N* = 14	
→ FII FI		10	
→ FI F0		2	
→ FII F0		1	
→ FIII FI		1	
**No conversion of horizontal** **furcation class**		*N* = 4	
FI = FI		2	7
FII = FII		2	2
F0 = F0		-	7
(**b**)			
Vertical furcation subclass ** (*n* = 18)	**T_0_**	**T_1_**	**T_final_**
	*N* = 18	*N* = 18	*N* = 16
A	2 (11%)	7 (39%)	7 (44%)
B	3 (17%)	11 (61%)	9 (56%)
C	13 (72%)	0 (0%)	0 (0%)
**Conversion of vertical furcation subclass**			
→ B A	./.	2	1
→ C B	./.	10	1
→ C A	./.	3	2
→ B C → A B	7	0	1 extraction 1

T_0_ = baseline, T_1_ = 1 year, T_final_ = up to 7 years; * horizontal furcation class 0-III (Hamp et al., 1975) [24]; ** vertical furcation subclass A, B, C (Tarnow and Fletcher, 1984) [25].

## Data Availability

The data that support the findings of this study are available from the corresponding author upon request.

## Supplementary Materials

The following supporting information can be downloaded at: https://www.mdpi.com/article/10.3390/jcm13010093/s1. Table S1: Results from final model for the change of radiographic bone level from T_0_ to T_final_.
