# Supplementary material for: Management of Teeth with Grade 3 Endo-Periodontal Lesions by Combined Endodontic and Regenerative Periodontal Therapy
Source: J Clin Med. 2023 Dec 23;13(1):93. doi: 10.3390/jcm13010093 (PMC10779476; doi:10.3390/jcm13010093)
Supplement: Supplementary file 1 [file jcm-13-00093-s001.zip › jcm-2723919-supplementary.pdf]

Table S1. Results from final model for the change of radiographic bone level from T_0 to $T_{final.}$

Fixed Effects	Estimate (SE)	*P*-value
**(Intercept)**	−2.30 (1.68)	0.18
rBl at T_0	0.64 (0.13)	<0.0001
BOP_{final}	−1.11 (0.05)	0.044
**R2:**	0.408	
